# An HMM-Based Comparative Genomic Framework for Detecting Introgression in Eukaryotes

**DOI:** 10.1371/journal.pcbi.1003649

**Published:** 2014-06-12

**Authors:** Kevin J. Liu, Jingxuan Dai, Kathy Truong, Ying Song, Michael H. Kohn, Luay Nakhleh

**Affiliations:** 1 Department of Computer Science, Rice University, Houston, Texas, United States of America; 2 Department of Ecology and Evolutionary Biology, Rice University, Houston, Texas, United States of America; 3 The State Key Laboratory for Biology of Plant Diseases and Insect Pests, Institute of Plant Protection, Chinese Academy of Agricultural Sciences, Beijing, China; Max-Planck-Institut für Informatik, Germany

## Abstract

One outcome of interspecific hybridization and subsequent effects of evolutionary forces is introgression, which is the integration of genetic material from one species into the genome of an individual in another species. The evolution of several groups of eukaryotic species has involved hybridization, and cases of adaptation through introgression have been already established. In this work, we report on PhyloNet-HMM—a new comparative genomic framework for detecting introgression in genomes. PhyloNet-HMM combines phylogenetic networks with hidden Markov models (HMMs) to simultaneously capture the (potentially reticulate) evolutionary history of the genomes and dependencies within genomes. A novel aspect of our work is that it also accounts for incomplete lineage sorting and dependence across loci. Application of our model to variation data from chromosome 7 in the mouse (*Mus musculus domesticus*) genome detected a recently reported adaptive introgression event involving the rodent poison resistance gene *Vkorc1*, in addition to other newly detected introgressed genomic regions. Based on our analysis, it is estimated that about 9% of all sites within chromosome 7 are of introgressive origin (these cover about 13 Mbp of chromosome 7, and over 300 genes). Further, our model detected no introgression in a negative control data set. We also found that our model accurately detected introgression and other evolutionary processes from synthetic data sets simulated under the coalescent model with recombination, isolation, and migration. Our work provides a powerful framework for systematic analysis of introgression while simultaneously accounting for dependence across sites, point mutations, recombination, and ancestral polymorphism.

## Author Summary

Hybridization is the mating between individuals from two different species. While hybridization introduces genetic material into a host genome, this genetic material may be transient and is purged from the population within a few generations after hybridization. However, in other cases, the introduced genetic material persists in the population—a process known as introgression—and can have significant evolutionary implications. In this paper, we introduce a novel method for detecting introgression in genomes using a comparative genomic approach. The method scans multiple aligned genomes for signatures of introgression by incorporating phylogenetic networks and hidden Markov models. The method allows for teasing apart true signatures of introgression from spurious ones that arise due to population effects and resemble those of introgression. Using the new method, we analyzed two sets of variation data from chromosome 7 in mouse genomes. The method detected previously reported introgressed regions as well as new ones in one of the data sets. In the other data set, which was selected as a negative control, the method detected no introgression. Furthermore, our method accurately detected introgression in simulated evolutionary scenarios and accurately inferred related population genetic quantities. Our method enables systematic comparative analyses of genomes where introgression is suspected, and can work with genome-wide data.

This is a *PLOS Computational Biology* Methods article.

## Introduction

Hybridization is the mating between species that can result in the transient or permanent transfer of genetic variants from one species to another. The latter outcome is referred to as introgression. Mallet [1] recently estimated that "at least 25% of plant species and 10% of animal species, mostly the youngest species, are involved in hybridization and potential introgression with other species." Introgression can be neutral and go unnoticed in terms of phenotypes but can also be adaptive and affect phenotypes. Recent examples of adaptation through hybridization include resistance to rodenticides in mice [2] and mimicry in butterflies [3]. Detecting regions with signatures of introgression in eukaryotic genomes is of great interest, given the consequences of introgression in evolutionary biology, speciation, biodiversity, and conservation [1]. With the increasing availability of genomic data, it is imperative to develop techniques that detect genomic regions of introgressive descent.

**Figure 1:**
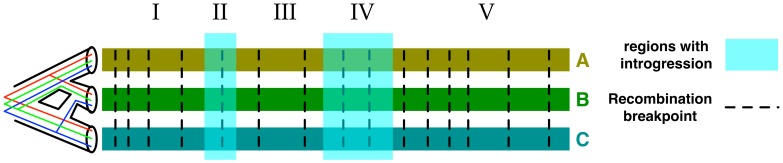
Evolutionary and genomic views of three genomes involving introgression. Hybridization between species B and C results in individuals of species B with genomes that are mosaics with regions of "vertical" descent from B and others of introgressive descent from C. Walking along the genomes from left to right, local genealogies are observed, and when a recombination breakpoint is crossed, the local genealogy changes. (Here, the term local genealogy refers to the local tree describing the evolutionary history of a single site in the alignment.) Switching of local genealogies of unlinked (broken by recombination) loci is known as incomplete lineage sorting (ILS). Further, the walk enters regions of introgressive descent (II and IV), where the genealogies switch due to hybridization. The complexity of the model stems from the co-occurrence of ILS and introgression, and the need to tease them apart. Within the phylogenetic network of the species (leftmost), three possible local genealogies are shown: one that agrees with how species split and diverged (red), one that is reflective of the introgression event (blue), and another that is a signature of ILS (brown).

Let us consider an evolutionary scenario where two speciation events result in three extant species A, B, and C, with A and B sharing a most recent common ancestor. Further, some time after the splitting of A and B, hybridization occurs between B and C (that is, sexual reproduction of individuals from these two species). This scenario is depicted by the phylogenetic network in Fig. 1. Immediately upon hybridization, approximately half of the hybrid individual's genome comes from an individual in species B, whereas the remainder comes from an individual in species C. However, in homoploid hybridization, where the hybrid offspring has the same ploidy level as the two parental species, hybridization is often followed by back-crossing (further mating between the hybrid population and either of the two parental populations). Repeated back-crossing, followed by the effects of genetic drift and natural selection, results in genomes in the hybrid individuals that are mosaics of genomic material from the two parental species, yet not necessarily with a 50–50 composition. Thus, detecting introgressed regions requires scanning across the genome and looking for signals of introgression.

In a comparative framework, detecting introgressed regions can be achieved by evolutionary analysis of genomes from the parental species, as well as genomes from introgressed individuals. In such an analysis, a walk across the genomes is taken, and local genealogies are inspected; incongruence between two local genealogies can be taken as a signal of introgression [4]. (Here, we focus on topological incongruence; see [5] for a related discussion on local variation of coalescence times.) However, in reality, the analysis is more involved than this, owing to potentially confounding signal produced by several factors, a major one of which is incomplete lineage sorting (ILS). As recombination breaks linkage across loci in the genome, the result is that independent loci might have different genealogies by chance, which is known as ILS. ILS is common to several groups of eukaryotic taxa where species diverged with insufficient time for all genomic loci to completely sort, resulting in a scenario where introgression and ILS effects need to be distinguished [3], [6]–. Fig. 1 illustrates this issue, where local genealogies across recombination breakpoints differ due to ILS, but also differ inside vs. outside introgressed regions. While other factors, such as gene duplication and loss [10], could potentially add to the complexity of the phylogenetic and genomic patterns, we focus here on introgression and ILS.

Recently, new methods were proposed to detect introgression in the presence of ILS. Durand *et al.*'s 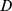 statistic allows for a sliding-window analysis of three-taxon data sets, while accounting for introgression and ancestral polymorphism [11]. However, this statistic assumes an infinite-sites model and independence across loci. Yu *et al.* [5] proposed a new statistical model for the likelihood of a species phylogeny model, given a set of gene genealogies, accounting for both ILS and introgression. However, this model does not work directly from the sequences; rather, it assumes that gene genealogies have been estimated, and computations are based on these estimates. Further, the model assumes independence across loci. Of great relevance to our work here is an array of hidden Markov model (HMM) based techniques that were introduced recently for analyzing genomic data in the presence of recombination and ILS [12]–[14]; however, these methods do not account for introgression. A recent extension [15] was devised to investigate the effects of population structure and migration. Finally, Saguaro is a recent method that combines HMMs with artificial neural networks to annotate genomic regions into different classes based upon local phylogenetic incongruence [16]. The classes are meant to categorize local genealogies, but the method is not aimed at elucidating the cause of incongruence.

In this paper, we devise a novel model based on integrating phylogenetic networks with hidden Markov models (HMMs). The phylogenetic network component of our model captures the relatedness across genomes (including point mutation, recombination, ILS, and introgression), and the HMM component captures dependence across sites and loci within each genome. Using dynamic programming algorithms [17] paired with a multivariate optimization heuristic [18], the model can be trained on genomic data, and allows for the identification of genomic regions of introgressive descent. We applied our model to chromosome 7 genomic variation data from three mouse data sets. Our analysis recovered an introgression event involving the rodenticide resistance gene *Vkorc1*, which was recently reported in the literature [2]. Based on the analysis, 9% of sites within chromosome 7 are in fact of introgressive origin, which is a novel finding in that previously only a localized region (that included *Vkorc1*) had been identified, with no further regions scanned. When applied to the negative control data set, our model did not detect any introgression, further attesting to its robustness. Our software is publicly available as part of the open-source PhyloNet distribution [19]. The method and software will enable new analyses of eukaryotic data sets where introgression is suspected, and will further help shed light on the Tree of Life—or, Network of Life.

## Materials and Methods

### Problem definition

**Figure 2:**
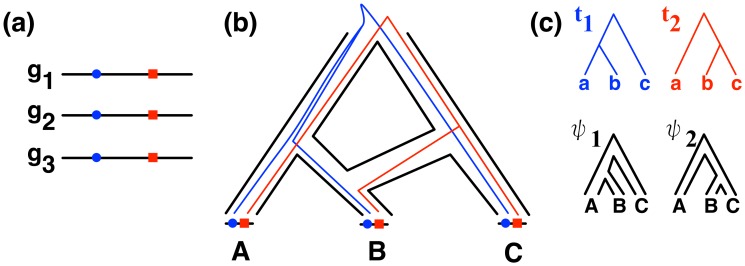
Local genealogies and parental species trees. The set 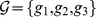 of genomes (a) have a reticulate evolutionary history, where individuals in B have some genetic material from the common ancestor of B and A, and other genetic material from C (b). In particular, the "blue locus" in the genomes has 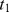 as its local genealogy and the "red locus" in the genomes has 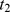 as its local genealogy (c). Further, genealogy 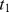 for the blue site evolved within the parental species tree 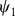, whereas genealogy 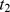 for the red locus evolved within the parental species tree 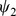.

Let 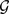 be a set of aligned genomes 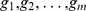, and 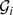 denote the 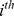 site in the alignment (if we view the alignment as a matrix where the rows are the genomes and the columns are the sites, then 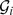 is the 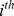 column in the matrix). Since the genomes are aligned, every 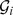 has evolved down a local genealogy, and since we assume that hybridization has occurred, each local genealogy has evolved within the branches of a parental tree. This is illustrated in Fig. 2.

It is important to note that for each 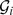, any tree could be the local genealogy. That is, if we denote by 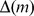 the set of rooted binary trees on 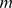 leaves, then for each 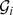, it is the case that 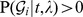, for every tree 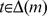 along with its branch lengths 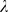. However, the set of parental species trees is always constrained by the actual evolutionary history of species. For example, in Fig. 2, only the two shown trees 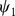 and 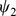 are the possible parental species trees. Given a set 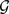 of 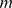 aligned genomes, each of length 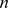, and a set 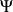 of parental species trees, we define a set of 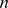 random variables 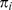 each of which takes values in the set 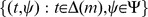. We are now in position to define the problem for which we provide a solution:

- **Input:** A set 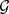 of 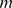 aligned genomes, each of length 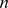, and a set 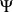 of parental species trees.
- **Output:** For each site 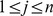, the probability

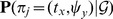
(1)for every 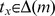 and 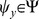.

Once this problem is solved and the method is run on a set of aligned genomes, we will be able to deduce the evolutionary history of every site, thus answering questions such as (1) which regions in the genomes are of introgressive descent (these would be the ones whose parental species tree, for the example in Fig. 2, is 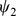; (2) is there recombination within introgressed regions (these would be indicated by switching among local genealogies in a region yet all genealogies evolved within 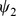); and, (3) what is the distribution of lengths of introgressed regions.

### The PhyloNet-HMM model: A simple case first

**Figure 3:**
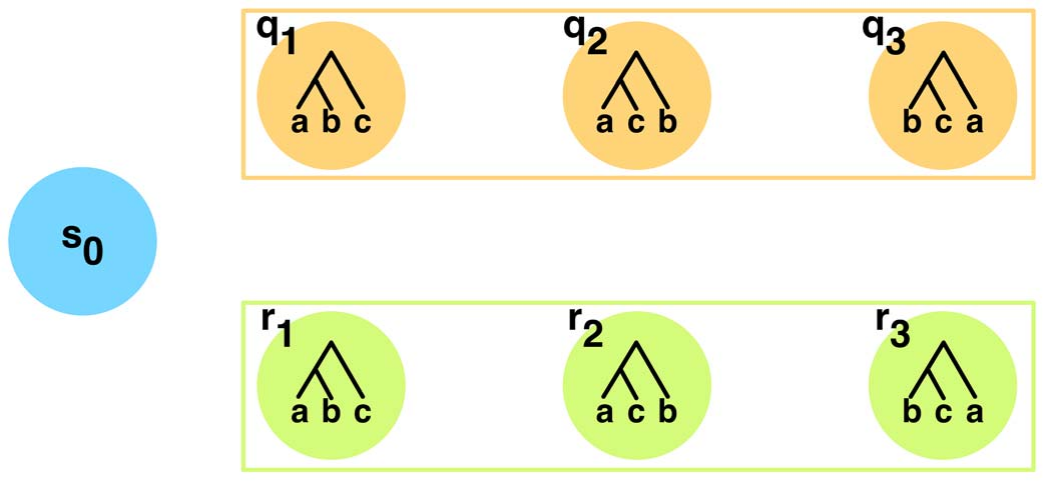
Illustrating PhyloNet-HMM. The structure of the HMM (only states are shown) that PhyloNet-HMM builds for the simple scenario of one individual sampled per species in Fig. 2. The three 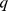 states correspond to genomic regions whose evolution follows the parental tree 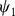, and there is a state for each of the three possible local genealogies. The three 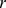 states correspond to genomic regions whose evolution follows the parental tree 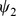, and there is a state for each of the three possible local genealogies. 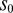 is the start state. See text for emission and transition probabilities.

Let us consider the scenario of Fig. 2, where only one individual is sampled per species. We propose a hidden Markov model (HMM) for modeling the evolution of the three genomes. The HMM for this simple case would consist of 7 states: a start state 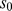, and six additional states: 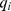 (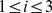), corresponding to three possible local genealogies within parental tree 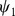, and 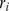 (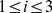), corresponding to three possible local genealogies within parental tree 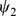. We denote by 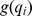 and 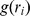 the local genealogies to which states 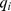 and 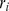 correspond, respectively; see Fig. 3.

In this model, transition between two 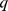 states or two 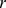 states corresponds to switching across recombination breakpoints. The probabilities of such transitions have to do with population parameters (e.g., population size, recombination rates, etc.). Transition from a 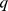 state to an 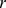 state indicates entering a introgressed region, while transition from an 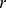 state to a 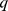 state indicates exiting an introgressed region. The probabilities of such transitions have to do, in addition, with introgression and evolutionary forces (back-crossing, selection, etc.). Each state emits a triplet of letters that corresponds to a column in the three-genome sequence alignment. The probability of emitting such a triplet can be computed using a standard phylogenetic substitution model [20].

Following the approaches of [12], [21], the transition probabilities in our model do not represent parameters in an explicit evolutionary model of recombination and introgression. Our choice was made to ease analytical representation and to permit tractable computational inference. We contrast our choice with alternative approaches: examples include (in order of increasing tractability of computational inference at the cost of more simplifying assumptions) methods incorporating the coalescent-with-recombination model [22], the sequentially Markovian coalescent-with-recombination model [14] (which adds the single assumption that coalescence cannot occur between two lineages that do not share ancestral genetic material), and the discretized sequentially Markovian coalescent-with-recombination model [23] (which additionally discretizes time).

Assuming that the probability of a site (or locus) in the genome of B being introgressed (in this case, inherited from C) is 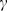, we follow the model of [5] and use this parameter to constrain the transition probabilities. Furthermore, we capture topological changes in local genealogies due to recombination using parameters 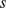
—the probability of switching from a local genealogy congruent with its containing parental tree to one that is incongruent—and 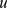
—the probability of switching from a gene genealogy incongruent with its containing parental tree to one that is congruent. Finally, we model incomplete lineage sorting by allowing every local genealogy with the probability of observing it given its containing parental tree [24].

For example, assume a site is emitted by state 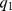 and consider the next site. If the next site is in an introgressed region, the HMM should switch, with probability 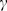, to an 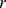 state. If the next site is not in an introgressed region, then the HMM should stay in the 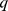 states, with probability 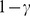, and the next HMM state depends upon whether or not the two sites are separated by a recombination breakpoint that causes a change in local genealogy incongruence (with respect to the containing parental tree 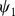): if they are, then the HMM should switch from state 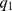 to a different state 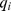 (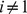) with probability 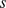; otherwise, the HMM should stay in state 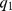 with probability 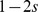. Thus, the transition probability from 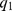 to any other 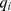 (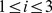) state is 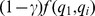 and to any 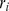 (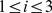) state is 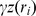, where 
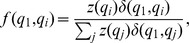




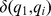
 is either 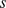 or 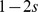 depending on whether or not the HMM transition corresponds to a change in local genealogy incongruence, 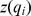 is the probability of genealogy 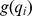
's topology given the parental tree in 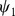, and 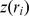 is the probability of genealogy 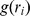
's topology given the parental tree 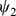. The 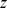 quantities are computed under the coalescent using the technique of [24].

If we denote by 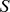 the set 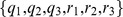 of (non-start) states, then a transition from the start state 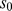 to a state 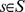 occurs according to the the normalized gene tree probability 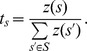



For 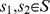 such that 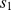 and 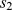 correspond to the same parental tree, let 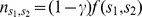. Furthermore, for 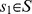, let 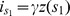. Then, the full transition probability matrix, with rows labeled 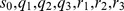 from top to bottom, and similarly for columns (from left to right), is
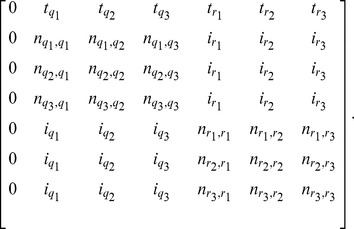



Given that 
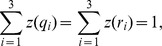



and 
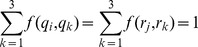
for every pair of indices 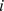 and 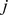, it follows that the entries in each row of the matrix add up to 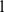. Further, the HMM always starts in state 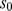; that is the initial state probability distribution is given by 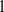 for state 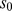 and 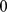 for every other state.

Once in a state 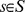, the HMM emits an observation 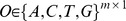, which is a vector in the genomic sequence alignment. Emissions occur according to a substitution model 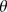 (we used the generalized time-reversible (GTR) model [25]), yielding the emission probability
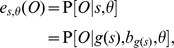



where 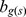 are the branch lengths of the gene tree associated with state 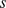. (It is straightforward to extend our model to other substitution models, including models nested within the GTR model and the GTR+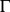 model, where 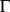 is an additional parameter for rate variation across sites.)

### The PhyloNet-HMM model: The general case

Modeling a phylogenetic network in terms of a set of parental trees fails for most cases [26]. For example, if two individuals are sampled from species B in Fig. 1, then one allele of a certain locus in one individual may trace the left parent (to C), while another allele of the same locus but in the other individual may trace the right parent (to A). Neither of the two parental trees in Fig. 3 can capture this case. Similarly, if one individual is sampled per species, but multiple introgression events occur or divergence events follow the introgression, the concept of parental trees collapses [5].

To deal with the general case—where multiple introgressions could occur, multiple individuals could be sampled, and introgressed species might split and diverge (and even hybridize again later) —we propose the following approach that is based on MUL-trees [5].

**Figure 4:**
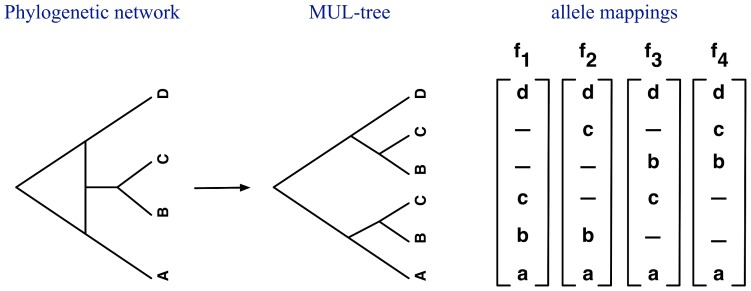
From a phylogenetic network to a MUL-tree. Illustration of the conversion from a phylogenetic network to a MUL-tree, along with all allele mappings associated with the case in which single alleles 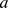, 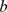, 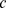 and 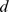 were sampled from each of the four species 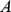, 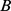, 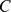 and 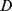, respectively.

The basic idea of the method is to convert the phylogenetic network 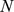 into a MUL-tree 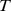 and then make use of some existing techniques to complete the computation on 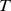 instead of on 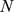. A MUL-tree [27] is a tree whose leaves are not uniquely labeled by a set of taxa. Therefore, alleles of individuals sampled from one species, say 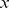, can map to any of the leaves in the MUL-tree 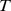 that are labeled by 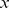. For network 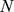 on taxa 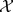, we denote by 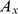 the set of alleles sampled from species 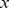 (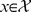), and by 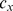 the set of leaves in 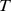 that are labeled by species 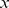. Then an *allele mapping* is a function 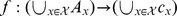 such that if 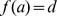, and 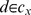, then 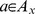 [5]. Fig. 4 shows an example of converting a phylogenetic network into a MUL-tree along with all allele mappings when a single allele is sampled per species. The branch lengths and inheritance probabilities 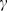 are transferred from the phylogenetic network to the MUL-tree in a straightforward manner (see [5] for details).

Now, two changes to the PhyloNet-HMM given for the simple case above are required. While in the simple case above, we used two classes of states (the 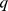 and 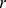 states), in the general case, the PhyloNet-HMM will contain 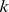 classes of states, where 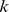 is the number of all possible allele mappings. As above, the transitions within a class of states corresponds to local phylogeny switching due to recombination and ILS, whereas transitioning between classes corresponds to introgression breakpoints. Second, the probability of observing a genealogy's topology given a containing parental tree is now computed using the method of [5], since the methods of [24], [28] are not applicable to MUL-trees.

### Learning the model and conducting inference

We used a hill-climbing heuristic to infer model parameters 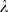 that maximize the likelihood of the model 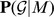. Here, the model 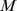 consists of

- the parental trees (topologies and branch lengths);
- local genealogies (topologies and branch lengths);
- the DNA substitution model parameters 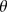;
- the parental tree switching probability, 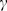; and.
- the parameters 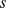 and 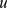, which contribute to local genealogy switching within a containing parental tree.

Notice that the 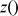 values are completely determined by the parental tree branch lengths and gene tree topology; hence, they are not free parameters in this model.

The standard forward and backward algorithms [17] were used to compute the model likelihood for fixed 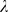. We used Brent's method [18] as a univariate optimization heuristic during each iteration of the hill-climbing search heuristic. To reduce the possibility of overfitting during optimization, branch length parameters were optimized for each topologically distinct parental tree, and similarly for each topologically distinct unrooted gene genealogy (since we use a reversible substitution model). States therefore "shared'' branch length parameters based on topological equivalence of parental trees and gene genealogies.

To evaluate the effectiveness of our optimization heuristic, we utilized different starting points for the model inference phase. We found that our heuristics were robust to the choice of starting point since the searches all converged to the same solution (data not shown). We found that the choice of starting point only affected search time.

After model parameter values were inferred, Viterbi's algorithm [17] was used to compute optimal state paths and, thus, annotations of the genomes. More formally, using Viterbi's algorithm, we computed 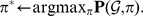



Further, we used the forward and backward algorithms to conduct posterior decoding and assess confidence for the states on a path 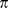: 
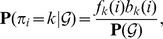



where 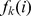 is the probability of the observed sequence alignment up to and include column 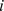, requiring that 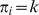 (computable with the forward algorithm); 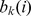 is the probability of the last 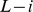 columns (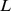 is the total number of columns in the alignment), requiring that 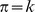 (computable with the backward algorithm); and, 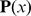 is the probability of the alignment (computable with either the forward or backward algorithms).

In the Results section, we show results based on both the optimal path, 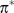, as well as posterior decoding, as the latter provides the probabilities in Eq. (1) in the problem formulation above.

### Simulated data

**Figure 5:**
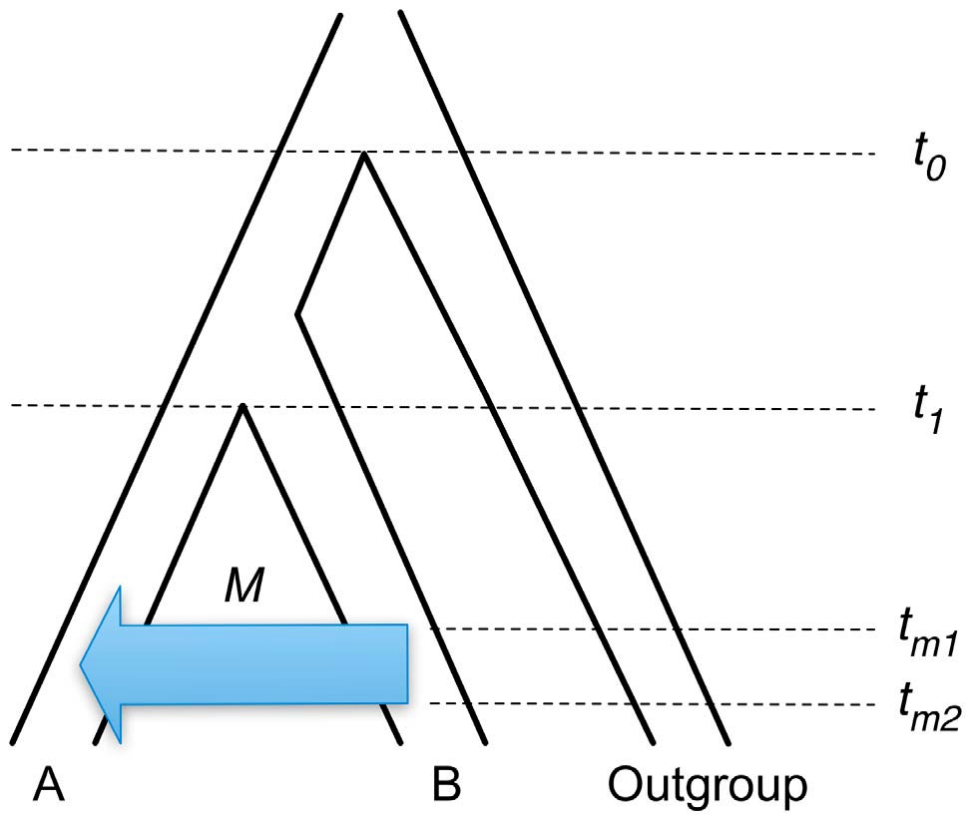
Model used for simulation of introgression. Migration from population B to population A proceeds at rate 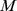, beginning at time 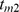 and ending at time 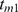. Times 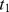 and 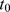 correspond to the split of populations A and B and the split of the outgroup population from the ancestral population of A and B, respectively.

To evaluate the performance of PhyloNet-HMM in scenarios where the true history of evolutionary events are known, we simulated data under the coalescent model [29] with recombination, isolation, and migration [22] using ms [30]. The specific model used for our simulation (Fig. 5) is based upon the consensus phylogeny for the species in our empirical study [31], to which we added migration processes. It is important to note that the model differs in one aspect compared to the one in the empirical study: the empirical data sets were sampled so that one *Mus musculus* sample had a very low chance of being introgressed, whereas both M samples in the simulation may be involved in introgression.

The simulation conditions were based upon consensus estimates from relevant prior literature (summarized in Table 1). We used a divergence time between in-group taxa of 1.5 Mya, generation time of 2 generations per year, and an effective population size 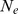 of 50,000, which implies divergence time 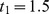 between the M and S populations. The outgroup population split from the ancestral population of A and B at time 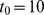. We used a cross-over rate 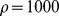, corresponding to 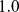 cM/Mb (compare with the 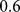 cM/Mb reported for mice and the 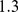 cM/Mb reported for humans [32]). We explored multiple migration scenarios hypothesizing either no migration (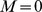) or migration at one of two different rates (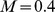 or 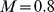). For scenarios including migration, we utilized two different sets of relatively recent migration times (either between 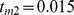 and 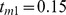 or between 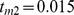 and 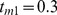) compared to the divergence time between A and B. Finally, substitutions occurred according to 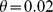, corresponding to 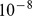 substitutions/site/year based on the 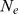 estimate above (compared with 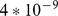 substitutions/site/year reported by [33]).

**Table 1:** Previously reported population genetic estimates upon which our simulation parameter settings were based.

Population genetic quantity	Estimate	Source
Divergence time to MRCA of *M. musculus* and *M. spretus*	At least 1.5 Mya bp	[31]
Number of *M. m. domesticus* generations per year	1–2	[41]
Number of *M. spretus* generations per year	2	[41]
*M. m. domesticus* effective population size	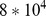 to 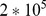	[42]
*M. m. domesticus* effective population size	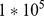 to 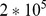	[43]
*M. m. domesticus* effective population size	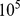	[41]
*M. m. domesticus* effective population size		
(using mutation rate estimate most similar to [33])	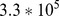 to 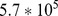	[44]

The branch lengths (in coalescent units) used for our simulation were based upon the previously reported quantities. See text for more details.

A simulation condition consisted of a setting for each simulation parameter (in 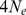 units, as required by ms [30]). For each condition, we repeated simulation to produce twenty replicate datasets per condition. The simulation of an individual dataset proceeded in two steps. First, ms was used to simulate local gene genealogies given the the coalescent model specified by the simulation condition. Then, using seq-gen [34], DNA sequence evolution was simulated on each local genealogy under the Jukes-Cantor model of substitution [35]. Sequences were simulated with total length of 100 kb distributed across the local genealogies.

### Mouse sample selection and data sets

Our study utilizes six mice that were either newly sampled or from previous publications. Details for the six mice are listed in Table 2.

**Table 2:** Mouse samples and data sets.

**Sample name**	**Species/ssp.**	**Alias**	
Spanish-mainland-domesticus	*M. m. domesticus*	MWN1287	
Georgian-domesticus	*M. m. domesticus*	DGA	
A-spretus	*M. spretus*	SPRET/EiJ	
B-spretus	*M. spretus*	SEG/Pas	
A-musculus	*M. m. musculus*	Yu2097m	
B-musculus	*M. m. musculus*	Yu2120f	
**Sample name**	**Origin**	**Gender**	**Source**
Spanish-mainland-domesticus	Roca del Valles, Catalunya, Spain	Female	[37]
Georgian-domesticus	Adjaria, Georgia	Male	[31], [37]
A-spretus	Puerto Real, Cadiz Province, Spain	Male	This study
B-spretus	Sante Fe, Granada Province, Spain	Male	[38]
A-musculus	Urumqi, Xinjiang, China	Male	[37]
B-musculus	Hebukesaier, Xinjiang, China	Female	[37]
**Data set**	**Set of samples used**		
*M. m. domesticus*	Spanish-mainland-domesticus, Georgian-domesticus, A-spretus, B-spretus		
*M. m. musculus* control	A-musculus, B-musculus, A-spretus, B-spretus		

We obtained a new mouse sample and also used existing mouse samples from previous studies. The array CEL files for existing mouse samples are available online (http://cgd.jax.org/datasets/diversityarray/CELfiles.shtml and by request from the authors of [38]). The introgression scans examined patterns of local phylogeny switching involving an *M. m. domesticus* sample from the region of sympatry with two *M. spretus* strains and a baseline *M. m. domesticus* sample from far away. The control scans utilized the two *M. spretus* strains along with two wild *M. m. musculus* mice that were known to not have introgressed with *M. spretus*.

Newly sampled mice were obtained as part of a tissue sharing agreement between Rice University and Stefan Endepols at Environmental Science, Bayer CropScience AG, D-40789 Monheim, Germany and Dania Richter and Franz-Rainer Matuschka at Division of Pathology, Department of Parasitology, Charité-Universitätsmedizin, D-10117 Berlin, Germany (reviewed and exempted by Rice University IACUC).

The *M. m. domesticus* data set was constructed as follows. We included a wild *M. m. domesticus* sample from Spain, part of the sympatry region (i.e., where the species co-occur geographically) between *M. m. domesticus* and *M. spretus*. To help maximize genetic differences as part of the design goals of our pipeline, we also selected a "baseline'' *M. m. domesticus* sample that originated from a region as far from the sympatry region as possible. Thus, we chose a mouse from the country of Georgia in Asia where *M. spretus* does not occur, and, presumably, *M. m. domesticus* there are ancestral to those *M. m. domesticus* that are part of derived populations in Western Europe, including Spain, and that encountered *M. spretus* during their westward dispersal. We utilized two *M. spretus* samples. The samples came from different parts of the sympatry region in Spain. The *M. m. musculus* control data set contained two wild *M. m. musculus* samples from China and the above two *M. spretus* samples.

The Mouse Diversity Array was used to obtain the empirical data used in our study [36]. Data for previously published samples were obtained from [31], [37], [38]. Since the probe sets in these studies differed slightly, we used the intersection of the probe sets in our study. A total of 535,988 probes were used.

We genotyped all raw reads using MouseDivGeno version 1.0.4 [38]. We utilized a threshold for genotyping confidence scores of 0.05. We phased all genotypes into haplotypes and imputed bases for missing data using fastPHASE [39]. Less than 15.1% of genotype calls were heterozygous or missing and thus affected by the fastPHASE analysis. The genotyping and phasing analyses were performed with a larger superset of samples. The additional samples consisted of the 362 samples used in [38] that were otherwise not used in our study. After genotyping and phasing was completed, we thereafter used only the samples listed in Table 2 in the Appendix.

Genomic coordinates and annotation in our study were based on the GRCm38.p2 reference genome (GenBank accession GCA_000001635.4). MouseDivGeno also makes use of data from the MGSCv37 reference genome (GenBank accession GCA_000001635.1).

## Results/Discussion

To assess confidence in our method's detection of regions of introgressive origin, we used a modified version of the posterior decoding. In our simulations as well as biological data analyses, there are 15 states corresponding to the "introgressed" parental tree: 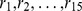. As we are interested in assessing confidence in whether a column 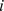 in the alignment 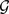 falls within an introgressed region, we computed for column 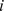 the quantity 
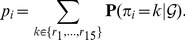
(2)


### Simulation study

We evaluated the performance of PhyloNet-HMM using simulated data sets. Here, we focus on results concerning inferred probabilities (computed using Eq. (2)) on simulations with different migration processes.

**Figure 6:**
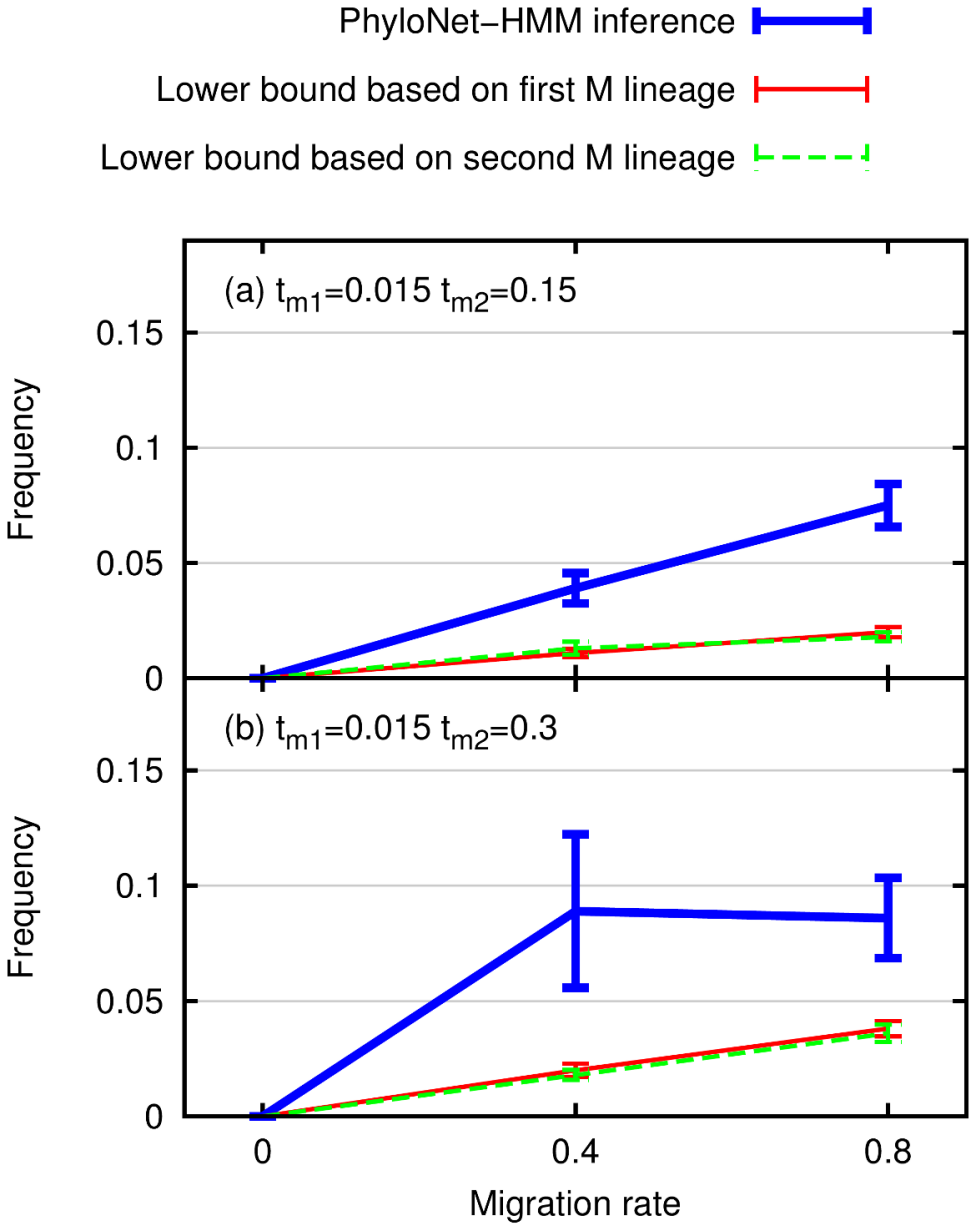
Comparison of the percentage of introgressed sites inferred by PhyloNet-HMM versus two lower bounds on simulated data sets. The percentage of sites is the number of sites 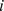 for which 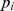, based on Eq. (2), is 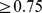, divided by the total number of sites in the simulated genomes, which is 100,000. The lower bounds on the true percentage of introgressed sites are based on the frequency that one of the two lineages from population A coalesced with lineages in population B between times 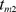 and 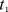. (See Materials and Methods for additional discussion.) Six model conditions are shown, encompassing three migration rates and two different dates of migration. A migration rate 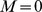 corresponds to a pure isolation model, whereas a migration rate 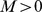 corresponds to an isolation-with-migration model. Standard error bars are shown, and the number of replicates for each model is 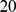.

In Fig. 6, we plot the percentage of sites 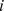 for which 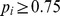 (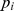 is computed using Eq. (2)) as a function of the migration rate. For the isolation-only model (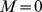), the method effectively infers no introgression for any of the sites. For the isolation-with-migration models (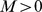), the inferred percentages of introgressed sites were greater than zero and increased as a function of the migration rate 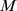. A potentially more informative comparison would be between the inferred percentages of introgressed sites and the percentages of sites in the simulation that involved migrant lineages. However, the simulation software that we used does not support annotating lineages in this way, nor is it a simple task to modify it to achieve this goal. (Furthermore, as noted above, we were unable to exactly simulate evolution under the evolutionary scenario in the empirical study since the simulation software did not permit us to constrain lineage evolution so that one of the samples from population A was not introgressed.)

On the other hand, for all simulated sites, the simulation software outputs the simulated gene genealogy under which the site evolved, along with branch lengths in coalescent units. This output from simulation can be used to obtain lower bounds on the true percentage of introgressed sites. Specifically, if a site evolved under a gene genealogy where one of the two A lineages and any subset of the B lineages are monophyletic and the lineages have a simulated coalescence time greater than 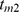 and smaller than 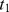, then migration must have occurred for those lineages to coalesce in that time span, based on the model used for simulation (Fig. 5). As shown in Fig. 6, for all simulated model conditions, the introgression frequency reported by PhyloNet-HMM is greater than or equal to lower bounds on the true introgression frequency, obtained using this observation.

Clearly, when the duration of the migration period increases, the variation in the estimates of our method increases, which results in a pattern that seemingly does not change from migration rate 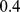 to 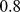. However, it is important to note that the extent of variability in this case precludes making a conclusion on the lack of increase in the percentage of sites. Nonetheless, the important message here is that the estimates of our method start varying more as the duration of the migration period increases.

We also found that the probability of observing a gene genealogy conditional on a containing parental tree differed between the two parental trees (results not shown). Under all simulation conditions, the inferred gene tree distribution (conditional on the containing parental tree) had multiple genealogies with non-trivial posterior decoding probabilities, suggesting that within-row transitions were capturing switching in local genealogies due to ILS. That is, the simulated data sets clearly had evidence of incongruence due to both introgression and ILS.

**Figure 7:**
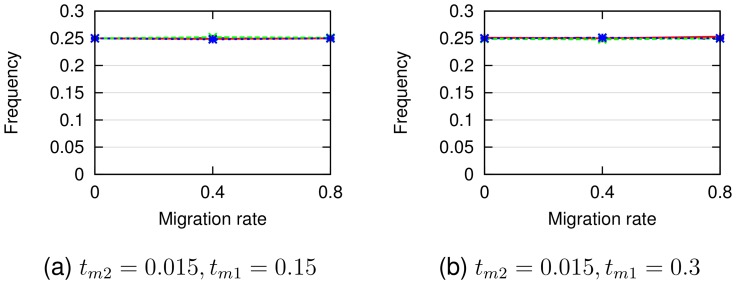
Empirical base frequencies inferred by PhyloNet-HMM on simulated data sets. Panels (a) and (b) show model conditions with migration times 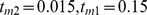 and 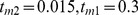, respectively, and different migration rates. Standard error bars are shown, and 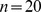.

**Figure 8:**
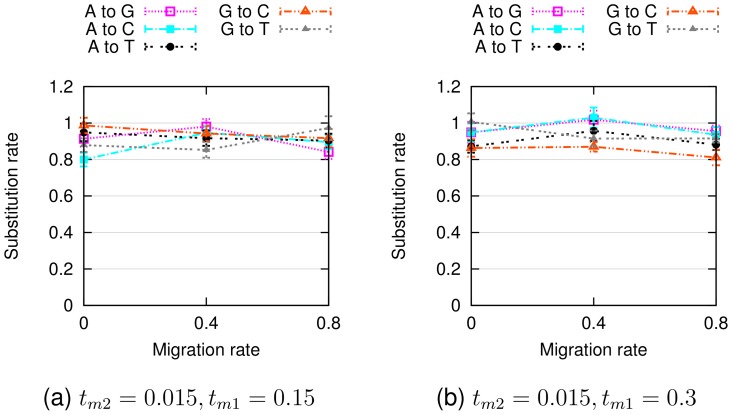
Empirical substitution rates inferred by PhyloNet-HMM on simulated data sets. Otherwise, figure layout and description match Fig. 7.

Finally, Fig. 7 and Fig. 8 show that in training our PhyloNet-HMM model on the simulated data, base frequencies were accurately estimated at 0.25 (which are the base frequencies for all four nucleotides we used in our simulations) and substitution rates were estimated generally between 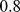 and 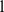 (we used 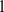 in our simulations). Further, the results were robust to the migration rates and durations of migration periods.

### Empirical study

**Figure 9:**
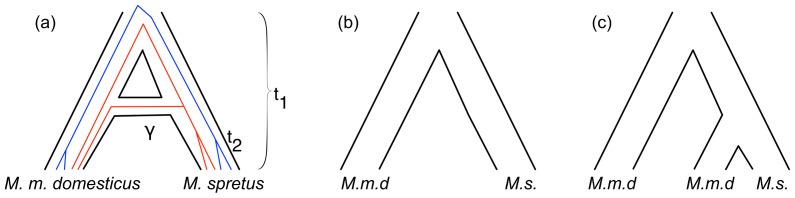
The phylogenetic network used in our analyses and the two parental trees. The phylogenetic network (a) captures introgression from *M. spretus* to *M. m. domesticus*. The red and blue lines illustrate two possible gene genealogies involving no introgression (blue) and introgression (red). The parental tree in (b) captures genomic regions with no introgression, while the parental tree in (c) captures genomic regions of introgressive descent.

We applied the PhyloNet-HMM framework to detect introgression in chromosome 7 in three sets of mice, as described above. Each data set consisted of two individuals from *M. m. domesticus* and two individuals from *M. spretus*. Thus the phylogenetic network is very simple, and has only two leaves, with a reticulation edge from *M. spretus* to *M. m. domesticus*; see Fig. 9(a). As we discussed above, the evolution of lineages within the species network can be equivalently captured by the set of parental trees in Fig. 9(b-c). Since in each data set we have four genomes, there are 15 possible rooted gene trees on four taxa. Therefore, for each data set, our model consisted of 15 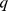 states, 15 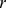 states, and one start state 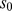, for a total of 31 states.

**Figure 10:**
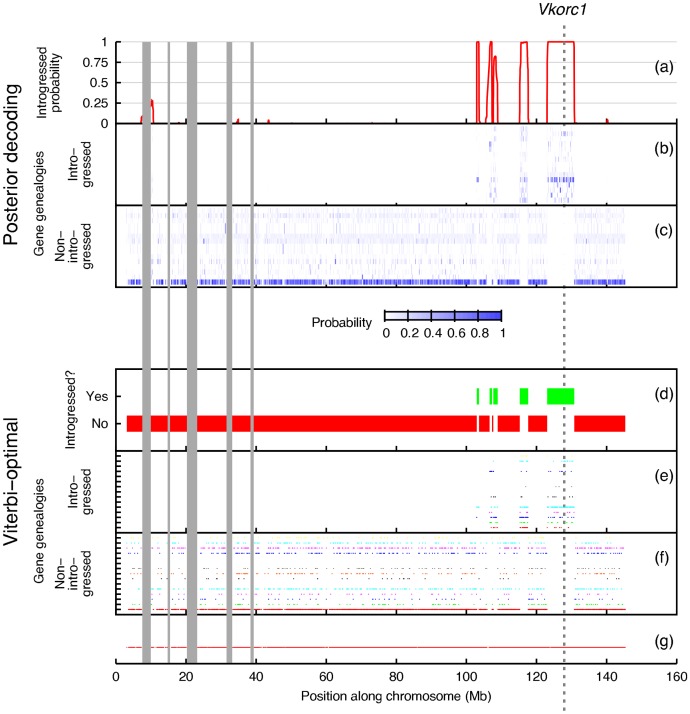
Introgression scans of chromosome 7 from the *Mus musculus domesticus* data set. Results in panels (a) through (c) are based on posterior decoding (Eq. 2). Panel (a) gives the probability that PhyloNet-HMM is in one of the introgressed (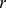) states. Panel (b) shows the probability that PhyloNet-HMM is in an introgressed (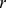) state corresponding to a particular gene genealogy, where each gene genealogy is displayed in a separate row and pixel intensity varies from white to blue to represent probabilities from 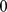 to 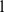. Panel (c) is identical to panel (b) except that non-introgressed (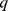) states are shown. Results in panels (d) through (f) are based upon a Viterbi-optimal trajectory. In panel (d), genomic regions are classified as having introgressed origin or not based on the hidden state that the Viterbi-optimal trajectory is in (either an 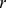 or 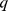 state, respectively). Panel (e) show the rooted gene genealogy inferred for each locus classified as introgressed in panel (d). Each distinct rooted gene genealogy is represented using a distinct color and row. Panel (f) shows the rooted gene genealogy inferred for the remaining loci (which were not classified as introgressed). Panel (g) shows loci sampled by the Mouse Diversity Array [36], which we used to genotype our samples. The dashed vertical line indicates the location of the *Vkorc1* gene, which was shown by [2] to be a driver gene in an introgression event between ( *M. m. domesticus* and *Mus spretus*) and leading to the spread of rodenticide resistance in the wild. The grey bars indicate regions with missing data that were approximately 100 kb or longer.

**Figure 11:**
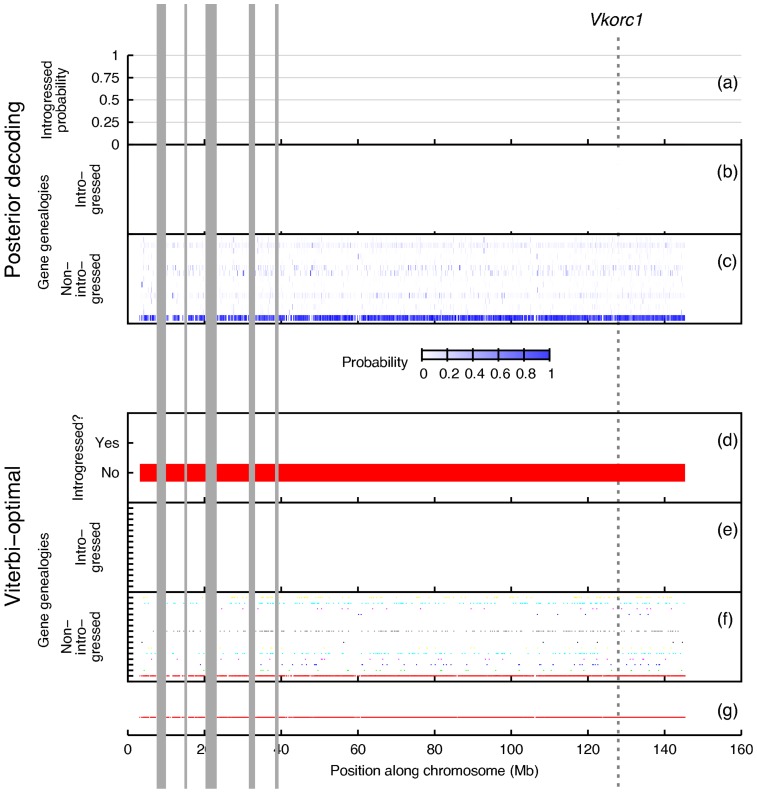
Introgression scans of chromosome 7 from the *Mus musculus musculus* data set. Figure layout and description are otherwise identical to Fig. 10.

We use our new model and inference method to analyze two types of empirical data sets. The first type includes individuals of known introgressed origin, and our model recovers the introgressed genomic region reported in [2] (Fig. 10). On the other hand, the second type consists of "control" individuals collected from geographically distant regions so as to minimize the chances of introgression (though, it is not possible to rule out that option completely). Our model detected no regions of introgressive descent in this dataset (Fig. 11).

We ran PhyloNet-HMM to analyze the *M. m. domesticus* data set, which consisted of samples from a putative hybrid zone between *M. m. domesticus* and *M. spretus* (Fig. 10). The data set covered all of chromosome 7, the chromosome containing the *Vkorc1* gene. *Vkorc1* is a gene implicated in the introgression event and the spread of rodenticide resistance in the wild [2].

Based on the pattern of recovered parental trees, the PhyloNet-HMM analysis detected introgression in the vicinity of the *Vkorc1* gene from approximately 123.0 Mb to 130.8 Mb, reproducing the findings of [2]. The presence of the introgression in the *M. m. domesticus* sample from mainland Spain but not the one from the country of Georgia suggests that the putative introgression may be polymorphic; preliminary results on additional Spanish samples (not shown) support this hypothesis. The analysis also uncovered recombination and incomplete lineage sorting in the region, as evidenced by incongruence among the rooted gene genealogies that were ascribed to loci.

The PhyloNet-HMM analysis detected introgression in 8.9% of sites in chromosome 7, containing over 300 genes. Notably, the analysis located similar regions in other parts of chromosome 7 which were not investigated by prior studies such as [2]. Examples include the region from 107.7 Mb to 108.9 Mb and the region from 115.2 Mb to 117.6 Mb. It is worth mentioning that the method does detect ILS within introgressed regions and outside those regions as well; yet, it does not switch back and forth between these two cases repeatedly (which is an issue that plagues methods that assume independence across loci).

As described by our model above, if we sum the transition probabilities from any 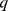 state to all 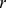 states, we obtain a value for 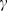. We performed this computation for each 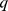 state, and took the average of all 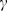 estimates based on each of the 15 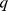 states. Our model estimates the value of 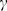 as 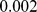. This can be interpreted as the probability of switching due to introgression, and can shed light on introgression parameters.

The posterior decoding probabilities, based on Eq. (2), for all positions in chromosome 7, are shown in Fig. 10(a). Clearly, the introgressed regions indicated by green bars in Fig. 10(d) have very high support (close to 1), particularly the region around the *Vkorc1* gene.

To further validate our approach, we repeated our scans on the *M. m. musculus* control data set (Fig. 11), which contained two sets of genomes of mice that are not known to hybridize. The first set of mice consisted of the *M. spretus* samples from the previous scan, and the second set of mice consisted of geographically and genetically distinct samples from *M. m. musculus*, which is not known to hybridize with *M. spretus* in the wild.

PhyloNet-HMM did not detect introgression on the control data set. The analysis recovered signatures of ILS, though, based on local incongruence among inferred rooted gene genealogies.

## Conclusions

In this paper, we introduced a new framework, PhyloNet-HMM, for comparative genomic analyses aimed at detecting introgression. Our framework allows for modeling point mutations, recombination, and introgression, and can be trained to tease apart the effects of incomplete lineage sorting from those of introgression.

We implemented our model, along with standard HMM algorithms, and analyzed an empirical data set of chromosome 7 from mouse genomes where introgression was previously reported. Our analyses detected the reported introgression with high confidence, and detected other regions in the chromosome as well. Using the model, we estimated that about 9% of the sites in chromosome 7 of an *M. m. domesticus* genome are of introgressive descent. Further, we ran an empirical analysis on a negative control data set, and detected no introgression. On simulated data, we accurately detected introgression (or the lack thereof) and related statistics from data sets generated under both isolation-with-migration and isolation-only models.

We described above how to extend the model to general data sets with arbitrary hybridization and speciation events, by using a MUL-tree technique. However, as larger (in terms of number of genomes) data sets become available, we expect the problem to become more challenging, particularly in terms of computational requirements. Furthermore, while the discussion so far has assumed that the set of states is known (equivalently, that the phylogenetic network is known), this is not the case in practice. This is a very challenging problem that, if not dealt with carefully, can produce poor results. In this work, we explored a phylogenetic network corresponding to a hypothesis provided by a practitioner. In general, the model can be "wrapped" by a procedure that iterates over all possible phylogenetic network hypotheses, and for each one the model can be learned as above, and then using model selection tests, an optimal model can be selected. However, this is prohibitive except for data sets with very small numbers of taxa. As an alternative, the following heuristic could be adopted instead: first, sample loci across the genome that are distant enough to guarantee that they are unlinked; second, use trees built on these loci to search for a phylogenetic network topology using techniques described in [40]; third, conduct the analysis as above. Of course, the phylogenetic network identified by the search might be inaccurate, in which case the use of an ensemble of phylogenetic networks that are close to that one in terms of optimality may be beneficial.
